# Genetic relationships between clinical and non-clinical strains of *Yersinia enterocolitica *biovar 1A as revealed by multilocus enzyme electrophoresis and multilocus restriction typing

**DOI:** 10.1186/1471-2180-10-158

**Published:** 2010-05-28

**Authors:** Sarita Mallik, Jugsharan S Virdi

**Affiliations:** 1 Microbial Pathogenicity Laboratory, Department of Microbiology, University of Delhi South Campus, Benito Juarez Road, New Delhi - 110 021, India

## Abstract

**Background:**

Genetic relationships among 81 strains of *Y. enterocolitica *biovar 1A isolated from clinical and non-clinical sources were discerned by multilocus enzyme electrophoresis (MLEE) and multilocus restriction typing (MLRT) using six loci each. Such studies may reveal associations between the genotypes of the strains and their sources of isolation.

**Results:**

All loci were polymorphic and generated 62 electrophoretic types (ETs) and 12 restriction types (RTs). The mean genetic diversity (*H*) of the strains by MLEE and MLRT was 0.566 and 0.441 respectively. MLEE (DI = 0.98) was more discriminatory and clustered *Y. enterocolitica *biovar 1A strains into four groups, while MLRT (DI = 0.77) identified two distinct groups. BURST (Based Upon Related Sequence Types) analysis of the MLRT data suggested aquatic serotype O:6,30-6,31 isolates to be the ancestral strains from which, clinical O:6,30-6,31 strains might have originated by host adaptation and genetic change.

**Conclusion:**

MLEE revealed greater genetic diversity among strains of *Y. enterocolitica *biovar 1A and clustered strains in four groups, while MLRT grouped the strains into two groups. BURST analysis of MLRT data nevertheless provided newer insights into the probable evolution of clinical strains from aquatic strains.

## Background

*Yersinia enterocolitica *is an important food- and water-borne gastrointestinal agent. It is known to cause a variety of syndromes ranging from mild gastroenteritis to more invasive diseases like terminal ileitis and mesenteric lymphadenitis mimicking appendicitis [1]. Blood transfusion associated septicaemia due to *Y. enterocolitica *has been reported to have high mortality [2]. Post infectious sequelae include reactive arthritis and erythema nodosum [1].

*Y. enterocolitica *is classified into six biovars (1A, 1B, 2, 3, 4 and 5) and more than 50 serotypes [3]. On the basis of pathogenicity, it has been grouped into highly pathogenic (biovar 1B), moderately pathogenic (biovars 2-5) and the so called non-pathogenic (biovar 1A) biovars. Recently, using comparative phylogenomics, Howard et al [4] suggested that these groups might represent three subspecies of *Y. enterocolitica*. The biovar 1A strains are quite heterogeneous serologically and have been isolated from a variety of sources *viz*. stools of diarrheic humans, animals, food and aquatic sources [5]. The biovar 1A strains are thought to be non-pathogenic as they lack pYV (plasmid for *Yersinia *virulence) plasmid and major chromosomal virulence determinants [1]. However, some biovar 1A strains are known to produce symptoms indistinguishable from that produced by the pathogenic biovars [6,7]. *Y. enterocolitica *biovar 1A has also been implicated in nosocomial [8] and food-borne [9] outbreaks. A serotype O:6,30 (biovar 1A) strain was reported to cause placentitis and abortion in pregnant ewes [10]. *Y. enterocolitica *biovar 1A was the most predominant biovar isolated from both livestock and humans during a survey in Great Britain in 1999-2000 and surely needs to be studied further [11]. Several recent studies suggest that these strains might possess novel, as yet unidentified, virulence determinants [12-16].

Serological heterogeneity notwithstanding, *Y. enterocolitica *biovar 1A has only limited genetic heterogeneity as revealed by different genotyping methods such as repetitive elements sequence-based PCR (rep-PCR) fingerprinting [17], 16S-23S intergenic spacer (IGS) region and *gyrB *restriction fragment length polymorphism [18], and multilocus variable number tandem repeat analysis (MLVA) [19]. Overall, these studies revealed presence of two clonal groups among biovar 1A strains. These studies also showed that clinical and non-clinical serotype O:6,30-6,31 (biovar 1A) strains clustered into two separate groups but failed to reveal any unequivocal associations between genotypes and the source of isolation.

Multilocus enzyme electrophoresis (MLEE) is an important tool used to study genetic relationships where allelic variations in housekeeping genes are indexed using electrophoretic mobilities of corresponding enzymes [20,21]. The technique has been used to study epidemiology of several pathogenic bacteria [22-26]. Multilocus restriction typing (MLRT), a recently developed tool, analyses restriction fragment length polymorphism of several housekeeping genes [27-29].

The objective of this study was to use MLEE and MLRT to gain further insight into the genetic heterogeneity and relationships among clinical and non-clinical strains of *Y. enterocolitica *biovar 1A.

## Methods

### Bacterial strains

Eighty one strains of *Y. enterocolitica *biovar 1A were examined in this study. Of these, sixty-five were isolated from clinical and non-clinical sources in India *viz*. diarrheic human patients (35), wastewater (18), swine (7) and pork (5) [30-32]. All isolates have been authenticated, and deposited with *Yersinia *National Reference Laboratory and WHO Collaborating Centre, Institut Pasteur, Paris (France). Of the remaining 16 isolates, ten were obtained from Elisabeth Carniel (*Yersinia *National Reference Laboratory and WHO Collaborating Centre, Institut Pasteur, Paris, France) and six from Jürgen Heesemann (Max von Pattenkofer Institute, Munich, Germany). *Y. enterocolitica *8081 (biovar 1B, serotype O:8), kindly provided by Mikael Skurnik (Haartman Institute, Finland) was used as the reference strain for both MLEE and MLRT.

The serotypes, sources of isolation, country of origin and reference laboratory accession numbers of these strains have been reported previously [17]. All strains were maintained as glycerol stocks at -40°C.

### Multilocus enzyme electrophoresis (MLEE)

The enzyme extracts were prepared as per the method described by Selander et al [20]. Briefly, cultures grown overnight in tryptone soy broth (TSB) were harvested by centrifugation at 10,000 *g *for 10 min at 4°C. The cells were washed twice in potassium phosphate buffer (0.15 M, pH 7.0) and the pellet was resuspended in 2 ml of buffer (10 mM Tris-HCl, 1 mM EDTA and 0.5 mM NADP, pH 6.8). The bacteria were lysed by sonication (Sonics) on ice and centrifuged at 13,000 *g *for 30 min at 4°C to obtain the supernatant (enzyme extract), which was stored in aliquots of 200 μl each at -40°C until use.

The enzyme extracts were subjected to horizontal gel electrophoresis in 0.9% (w/v) agarose and stained for specific enzyme activities according to the procedures described by Selander et al [20]. The enzymes studied were: malate dehydrogenase (MDH; EC 1.1.1.37), malic enzyme (ME; EC 1.1.1.40), glucose-6-phosphate dehydrogenase (G6P; EC 1.1.1.49), isocitrate dehydrogenase (IDH; EC 1.1.1.42), alpha esterase (EST-A; EC 3.1.1.1) and glutamate dehydrogenase (GD2; EC 1.4.1.4). The enzymes MDH, ME, G6P and IDH were electrophoresed in Tris citrate buffer (pH 8.0). For EST-A, potassium phosphate buffer (gel buffer, pH 7.0; electrode buffer, pH 6.7) was used and GD2 was electrophoresed in a lithium hydroxide buffer (gel buffer, pH 8.3; electrode buffer, pH 8.1).

Replicate samples from reference strain were run on each gel, which facilitated comparison of the gels. The mobilities of the enzymes from different samples on the same gel were compared. For each enzyme, the distinct mobility variants were designated as electromorphs and numbered in order of decreasing rate of anodal migration. The electromorphs of an enzyme were equated with alleles at the corresponding structural gene locus. Each strain was characterized on the basis of combination of its electromorphs obtained for the six enzymes. Distinct profiles of electromorphs corresponding to multilocus genotypes were designated as electrophoretic types (ETs).

### Statistical analyses

Computer programs written by Prof T. S. Whittam were used to analyze the ET data and calculation of genetic diversity [20]. Genetic diversity (*h*) at an enzyme locus (*i.e*., the probability that two isolates differ at the *j *locus) was calculated from the allele frequencies as *h*_*j *_= *n *(1 - Σ*x*_*i*_^2^)/*n *- 1), where *x*_*i *_is the frequency of the *i*th allele at the *j *locus and *n *is the number of isolates [33]. Mean genetic diversity per locus (*H*) was calculated as the arithmetic average of *h *values for all loci. The genetic distances between pairs of ETs were calculated as the proportions of loci at which dissimilar electromorphs occurred. Clustering of data was performed from a matrix of pairwise genetic distances by the average-linkage method (unweighted pair group method using arithmetic averages or UPGMA).

### Multilocus restriction typing (MLRT)

Genomic DNA was extracted using DNeasy tissue kit (Qiagen) as per the manufacturer's instructions. The six genes encoding housekeeping enzymes: malate dehydrogenase (*mdh*), adenylate cyclase (*cya*), glutamine synthetase (*glnA*), glucose-6-phosphate dehydrogenase (*zwf*), isocitrate dehydrogenase (*icdA*) and glutamate dehydrogenase (*gdhA*) were selected. For amplification of these genes, *Yersinia *consensus primers were designed using nucleotide sequences from *Y. enterocolitica *8081 (biovar 1B, AM286415), *Y. pestis *(AE009952) and *Y. pseudotuberculosis *(BX936398) available at EMBL and GenBank databases, after pairwise alignment of the sequences using ClustalW http://www.ebi.ac.uk/clustalW. Primers were designed with PRIMER SELECT software (DNAStar), and synthesized from Microsynth. The details of the primers are given in Table 1.

**Table 1:** Details of primers and restriction enzymes used for multilocus restriction typing (MLRT) of *Y. enterocolitica *biovar 1A

Target gene	Primer	Position*	Sequence (5'-3')	Annealing temperature	Amplicon size (bp)	Restriction enzyme	Restriction fragments (bp)†
*mdh *(malate dehydrogenase)	Mdh1 Mdh2	484705...484726 485301...485280	TAT ATG ACA TCG CGC CAG TGA C CAG CTT GCC CCA TAG ACA GAG T	61°C	597	*Hae*III *Rsa*I	102, 164, 331 179, 191, 227
*cya *(adenylate cyclase)	AdC1 AdC2	224199...224222 225200...225181	AAC CGC CTG CAA AAG AAA TGT AGT CCA GCC CGG ACG GTT AGC AC	66°C	1,002	*Hae*III *Sau96*I	22, 157, 346, 477 24, 128, 216, 634
*glnA *(glutamine synthetase)	GN1 GN2	36808...36830 37528...37506	TTC CGG TGG CAA GTC ATA CAG GT CAA ATA CGA AGG CGG CAA CAA AG	65°C	721	*Bgl*I *Sau96*I	70, 651 39, 85, 237, 360
*zwf *(glucose-6-phosphate dehydrogenase)	G6P1 G6P2	2570039...2570061 2570679...2570659	CCT GAA TAC CGC GCA TCG TCT CT AGG GCG CTG GGG CTA TTT TGA	65°C	641	*Rsa*I *BstN*I	32, 62, 109, 189, 249 128, 243, 376
*icdA *(isocitrate dehydrogenase)	IDH1 IDH2	1923868...1923889 1925035...1925014	GCG CTG AAG GAG AGG TTG ATG G CGC CTT CGG TGC CTT TGA TAA T	57°C	1,168	*Hae*III *Rsa*I	136, 185, 365, 480 125, 127, 221, 304, 391
*gdhA *(glutamate dehydrogenase)	GmD1 GmD2	4416077...4416094 4416600...4416579	GGG CAA AGG CGG CTC TGA TAC GTT CGC GGC ATA ATC TTC	66°C	524	*Hae*III *Mse*I	11, 42, 141, 320 21, 50, 121, 432

*: Reference strain *Y. enterocolitica *subspecies *enterocolitica *8081 (biovar 1B, serotype O:8), accession no. AM286415.

†: Restriction fragments of amplicons obtained for reference strain.

Polymerase chain reactions were performed in 25 μl of reaction mixture containing 1 × PCR buffer (10 mM Tris-HCl pH 8.8, 50 mM KCl, 0.1% Triton X-100, 1.5 mM MgCl_2_), 200 μM of each dNTP (MBI Fermentas), 20 pmoles each of forward and reverse primers, 2 U DyNAzyme™ II DNA polymerase (Finnzymes) and 100 ng of template DNA. All amplifications were performed in a PTC-100™ thermal cycler (MJ Research) according to the following cycling conditions: initial denaturation for 5 min at 94°C, 30 amplification cycles each consisting of 1 min denaturation at 94°C, annealing for 45 s at the temperatures as given in Table 1, and 1 min elongation at 72°C. The final extension was carried out at 72°C for 10 min. 5 μl of the PCR product was electrophoresed in 1% (w/v) agarose gel containing 0.5 μg ml^-1 ^ethidium bromide (EtBr) at 80 V for 1 h in 1 × Tris-acetate EDTA buffer (1 × TAE: 40 mM Tris acetate, 1 mM EDTA, pH 8.0). The 100 bp DNA ladder (New England Biolabs) served as the molecular size marker.

The restriction enzymes for MLRT were selected by an *in silico *restriction analysis of respective gene sequences of *Y. enterocolitica *8081 (biovar 1B) available in GenBank using MapDraw (DNAStar) such that polymorphism in the restriction sites was revealed. The PCR amplicons of six genes for all the 81 strains were digested with enzymes as shown in Table 1. Restriction digestion was carried out overnight at 37°C in 25 μl reaction mixture containing 8 μl of the PCR amplicon, 2.5 μl of 10 × buffer and 2 U of restriction enzyme (New England Biolabs). Restriction digests were analyzed by agarose gel electrophoresis (2.5% gel containing 0.5 μg ml^-1 ^EtBr in 1 × TAE buffer). Gels were run at 60 V and photographed under UV transillumination. The 50 bp and 100 bp DNA ladders (New England Biolabs or MBI Fermentas) served as the molecular weight standards.

The restriction patterns for all the isolates were analyzed using Diversity Database Software (version 2, Bio-Rad). Distinct restriction patterns for each locus were considered to represent separate alleles, and each allele was assigned a numeral. As with MLEE, the combination of alleles at each of the six loci gave a restriction type (RT). Strains were considered different if the allele of any of the six loci differed. The genetic diversity *h *was calculated as described for MLEE. The restriction profile for each isolate was entered into a database and used to construct a phylogenetic tree based on unweighted-pair group method with average (UPGMA) linkage of distance, using the START (Sequence Type Analysis and Recombination Tests) software package http://outbreak.ceid.ox.ac.uk/software.htm. In addition, clonal complexes within 81 biovar 1A strains were investigated using the BURST (Based Upon Related Sequence Types) algorithm of START software package.

### DNA sequencing and analysis

For each allele identified for the six genes used in MLRT, one amplicon was sequenced to confirm its identity. PCR products were purified with the QIAquick gel extraction kit (Qiagen) and DNA sequencing was performed by the Big-Dye terminator kit using an automated DNA sequencer (ABI PRISM 3730 genetic analyzer).

### Linkage disequilibrium analysis

Linkage disequilibrium for MLEE and MLRT data was calculated on the basis of the distribution of allelic mismatches between pairs of bacterial isolates among all the loci examined. The ratio of the variance observed (*V*_O_) in mismatches to the variance expected (*V*_E_) at linkage equilibrium provides a measure of multilocus linkage disequilibrium and can be expressed as the index of association (*I*_A_) as: *I*_A _= *V*_O_/*V*_E _- 1 [34,35]. For populations in linkage equilibrium, *V*_O _= *V*_E _and *I*_A _is not significantly different from zero, whereas values of *I*_A _significantly greater than zero indicate that recombination has been rare or absent. To determine whether *V*_O _was significantly different from *V*_E _in any sample, a Monte Carlo procedure was iterated, wherein alleles are repeatedly scrambled to eliminate any effect of linkage disequilibrium [36]. The LIAN version 3.5 software program [37] was used to calculate *I*_A _and standardized *I*_A _(*I*^S^_A_) values and perform Monte Carlo procedure.

### Calculation of diversity index

Simpson's diversity index (DI), a measure of the discriminatory ability of a given typing method, was calculated for MLEE and MLRT as described by Hunter and Gaston [38].

### Nucleotide sequence accession number

The nucleotide sequence data of six genes used in MLRT study reported in this paper have been deposited in GenBank database under the accession numbers FJ899547-FJ899554 and GQ229153-GQ229162.

## Results

### Electrophoretic types (ETs) and genetic diversity

Activities of six enzymes were detected in all 81 strains of *Y. enterocolitica *biovar 1A and the reference strain *Y. enterocolitica *8081. All enzyme loci studied were polymorphic and the number of alleles ranged from three (isocitrate dehydrogenase) to fifteen (glucose-6-phosphate dehydrogenase) (Table 2). The mean number of alleles per locus was 7.5. Esterase was the most polymorphic (*h *= 0.827), while glutamate dehydrogenase was the least polymorphic locus (*h *= 0.250). The mean genetic diversity (*H*) of all strains was 0.566 ± 0.088. Among the 81 *Y. enterocolitica *biovar 1A strains, 62 ETs (electrophoretic types) were identified. The reference strain *Y enterocolitica *8081 formed a distinct ET, ET63 (Table 3). Fifty seven ETs were represented only once in the data set. The ETs which were represented more than once were ET1 (ten isolates), ET8 (six isolates), ET6 (three isolates), and ETs 20, 36 and 42 (two isolates each).

**Table 2:** Genetic diversity at six enzyme loci in *Y. enterocolitica *biovar 1A

Technique	Enzyme locus	No. of alleles	Genetic diversity (*h*)
MLEE	MDH	4	0.490
	ME	6	0.637
	G6P	15	0.759
	IDH	3	0.438
	EST-A	12	0.827
	GD2	5	0.250
	Mean	7.5	0.566 ± 0.088 (*H*)
			
MLRT	*mdh*	5	0.481
	*cya*	3	0.355
	*glnA*	3	0.474
	*zwf*	3	0.644
	*icdA*	2	0.336
	*gdhA*	3	0.355
	Mean	3.2	0.441 ± 0.048 (*H*)

*H*: Mean genetic diversity.

**Table 3:** Details of electrophoretic types (ETs) and restriction types (RTs) of *Y. enterocolitica *biovar 1A

ET	N	Alleles at enzyme locus						Serotype (*n*)	Source	Country	RT	Profile*
		MDH	ME	G6P	IDH	EST-A	GD2					
1	10	2	3	6	2	12	3	O:6,30-6,31 (3), O:6,30 (6) O:7,8-8-8,19	Human (9) Pork	India India	1 1	312222 312222
2	1	2	3	13	2	12	3	O:6,30-6,31	Human	India	1	312222
3	1	2	3	14	2	12	3	O:6,30	Human	India	1	312222
4	1	2	3	7	2	12	3	ND	Pig throat	India	1	312222
5	1	2	3	7	2	4	3	O:41-43	Wastewater	India	1	312222
6	3	2	3	6	2	11	3	O:6,30-6,31 (2), O:6,30	Human	India	1	312222
7	1	2	4	7	2	4	3	O:6,30	Human	Ger	4	212122
8	1	2	4	6	2	4	3	O:6,30-6,31	Human	India	1	312222
9	1	2	4	2	2	12	3	O:6,30	Human	India	1	312222
10	1	2	4	6	2	12	3	O:6,30-6,31	Human	Fra	1	312222
11	1	3	4	5	2	12	3	NAG	Human	India	1	312222
12	1	3	3	5	2	12	3	O:6,30	Human	India	5	313222
13	1	4	3	6	2	4	3	O:41-42	Wastewater	India	6	213222
14	1	4	3	3	2	4	3	O:6,30	Human	India	1	312222
15	1	4	3	6	2	12	3	O:6,30	Human	India	1	312222
16	1	4	3	7	2	7	3	O:6,30	Human	India	1	312222
17	1	4	3	7	2	2	3	O:6,30	Human	India	1	312222
18	6	2	3	6	2	3	3	O:6,30-6,31	Wastewater	India	7	313122
								O:6,30-6,31 (2), O:10-34, NAG	Wastewater	India	2	312122
								NAG	Human	India	2	312122
19	1	2	3	9	2	3	3	O:6,30-6,31	Wastewater	India	2	312122
20	2	2	3	7	2	3	3	O:6,30-6,31	Wastewater	India	2	312122
21	1	2	3	7	2	8	3	O:7,8-8-8,19	Pork	India	3	521333
22	1	2	3	6	2	5	3	O:6,30	Human	India	1	312222
23	1	2	3	9	2	6	3	O:6,30	Human	Fra	4	212122
24	1	2	3	6	1	3	3	O:6,30-6,31	Wastewater	India	2	312122
25	1	2	3	7	3	3	3	O:6,30	Human	India	3	521333
26	1	3	3	4	2	3	3	O:6,30	Human	Fra	2	312122
27	1	3	3	9	2	6	3	O:10-34	Human	Fra	3	521333
28	1	2	5	2	2	8	3	O:10-34	Wastewater	India	3	521333
29	1	2	5	7	2	3	3	NAG	Pork	India	6	213222
30	1	2	5	7	2	1	3	O:7,8-8-8,19	Pork	India	1	312222
31	1	2	2	6	2	3	3	NAG	Human	India	2	312122
32	1	2	2	6	2	3	5	O:10-34	Wastewater	India	10	331222
33	1	2	4	11	2	3	3	NAG	Human	India	2	312122
34	1	2	4	13	2	3	3	ND	Pig throat	India	2	312122
35	1	2	4	7	2	10	3	O:6,30	Human	Ger	4	212122
36	2	2	4	7	2	8	3	O:6,30 ND	Human Pig throat	India India	3 3	521333 521333
37	1	2	4	7	2	8	3	NAG	Human	India	2	312122
38	1	2	4	9	3	3	3	ND	Pig throat	India	3	521333
39	1	2	4	15	3	3	3	ND	Pig throat	India	3	521333
40	1	2	4	12	3	8	3	O:7,8-8-8,19	Pork	India	3	521333
41	1	2	2	10	3	8	3	O:10-34	Wastewater	India	11	421333
42	2	2	4	6	1	3	3	NAG	Human	India	2	312122
43	1	2	4	4	1	3	3	O:6,30	NK	NK	4	212122
44	1	2	4	4	1	8	3	NK	Pig throat	India	1	312222
45	1	3	4	4	2	4	2	O:6,30	Human	Ger	2	312122
46	1	3	4	5	2	2	2	O:6,30	Human	Fra	2	312122
47	1	2	4	9	2	2	1	O:6,30	NK	NK	2	312122
48	1	2	4	9	3	6	2	O:10-34	Human	Fra	3	521333
49	1	5	3	6	2	12	2	O:15	Wastewater	India	5	313222
50	1	1	3	9	2	6	2	O:10-34	Human	Fra	3	521333
51	1	1	5	1	3	8	3	O:6,30-6,31	Human	India	3	521333
52	1	1	5	9	3	8	3	NAG	Wastewater	India	3	521333
53	1	3	5	7	3	8	3	NAG	Human	India	3	521333
54	1	2	5	8	3	8	3	O:6,30	Human	India	8	523333
55	1	1	5	7	2	8	4	O:10-34	Wastewater	India	3	521333
56	1	3	2	5	1	5	3	O:7,8-8-8,19	Pig throat	India	5	313222
57	1	3	2	6	1	6	3	O:6,30-6,31	Human	USA	12	312124
58	1	3	4	6	1	11	3	NAG	Human	India	1	312222
59	1	3	1	6	1	9	3	O:5	Human	NK	2	312122
60	1	2	4	6	1	12	5	O:6,30-6,31	Human	Fra	1	312222
61	1	3	3	9	1	12	5	O:6,30-6,31	Human	Fra	1	312222
62	1	2	6	6	1	1	5	O:6,31	Wastewater	India	9	613122
63	1	2	2	1	3	12	2	O:8	Patient	USA	13	111111

ET: Electrophoretic type; N: Number of strains with particular ET; MDH: malate dehydrogenase; ME: malic enzyme; G6P: glucose-6-phosphate dehydrogenase; IDH: isocitrate dehydrogenase; EST-A: alpha esterase; GD2: glutamate dehydrogenase.

*n*: Number of strains; NAG: non-agglutinable; ND: not determined; NK: not known

Ger: Germany; Fra: France

RT: Restriction type

*: Allele profile of genes in order *mdh, cya, glnA, zwf, icdA, gdhA*.

**Figure 1:**
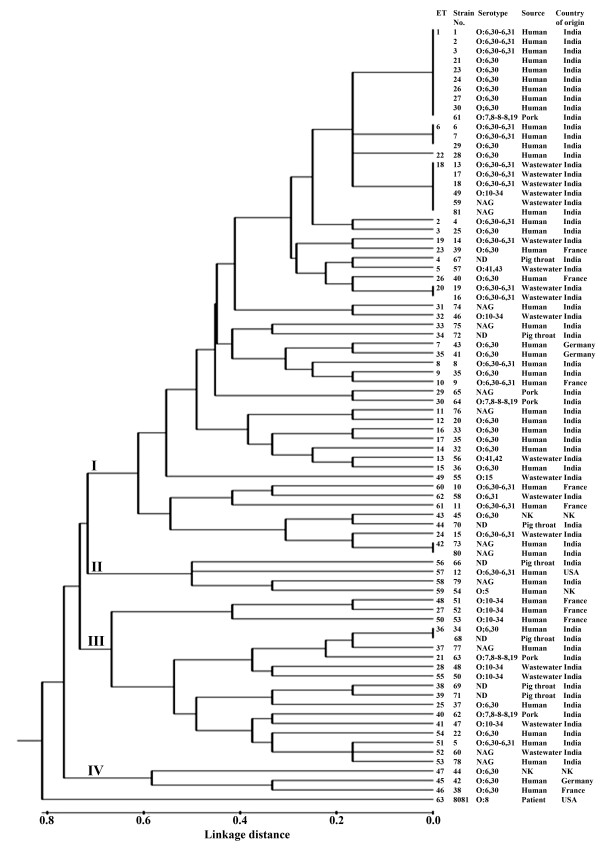
**UPGMA dendrogram showing genetic relationships among 62 electrophoretic types (ETs) of *Y. enterocolitica *biovar 1A**. NAG: non-agglutinable, ND: not determined, NK: not known.

The genetic relationships among strains of *Y. enterocolitica *biovar 1A as revealed by cluster analysis using UPGMA are shown in Figure 1. The shortest genetic distance (0.167) between the ETs corresponded to a single locus difference. The strains were grouped into 4 groups (I to IV) diverging at genetic distance of 0.76. The group I comprising 38 ETs (ET1-20, 22-24, 26, 29-35, 42-44, 49, 60-62) was the largest with 56 isolates belonging to different serotypes and sources. This group was highly diverse with several subclusters. This group also contained the most common ET, ET1 which was represented by 9 clinical isolates belonging to serotypes O:6,30-6,31 (3 isolates) and O:6,30 (6 isolates), and one pork isolate of serotype O:7,8-8-8,19. Another ET, ET18 was also predominant and contained 6 Indian strains which included three wastewater serotype O:6,30-6,31 isolates, one wastewater serotype O:10-34 isolate and two NAG isolates one each of aquatic and clinical source. Group II included 4 ETs (ET56-59) containing one pig throat isolate and 3 clinical isolates. Group III was formed by 18 isolates representing 17 ETs (ET 21, 25, 27, 28, 36-41, 48, 50-55). These strains belonged to diverse serotypes and sources from India (15 isolates) and France (3 isolates). The three French isolates formed a separate subgroup at a genetic distance of 0.64. Group IV included three European clinical serotype O:6,30 isolates representing ETs 45-47. MLEE dendrogram revealed that ET1 and ET36 represented by multiple isolates showed close association (linkage distance = 0.0) between isolates from pork/pig throat and human.

### Multilocus restriction typing

PCR amplicons were obtained for all six loci using primers and PCR conditions given in Table 1. For each of the six loci, PCR amplicons of respective sizes were obtained for all the 81 strains of *Y. enterocolitica *biovar 1A. The amplicons were digested with restriction enzymes as shown in Table 1. The RFLP profiles for each of the six loci are given in Additional file 1. Collating the PCR-RFLP data for six loci in 81 strains, 12 restriction types (RTs) were identified (Table 3). Reference strain *Y. enterocolitica *8081 (biovar 1B, serotype O:8) was represented by a distinct RT, RT13. RT1 was the most common restriction type and was present among 31 (37%) isolates. The second commonest type was RT2, represented by 20 (25%) isolates while RT3 was the third commonest (15 isolates, 19%) restriction type. Reproducibility of MLRT was checked by repeating RFLP using selected isolates. Same allelic profiles were obtained indicating reproducibility of MLRT.

The number of alleles present per locus and genetic diversity among 81 strains of *Y. enterocolitica *biovar 1A as determined by MLRT are given in Table 2. Glucose-6-phosphate dehydrogenase (*zwf*) locus was the most diverse (*h *= 0.644) while isocitrate dehydrogenase (*icdA*) was least diverse (*h *= 0.336). The mean genetic diversity (*H*) of all isolates was 0.441 ± 0.048.

**Figure 2:**
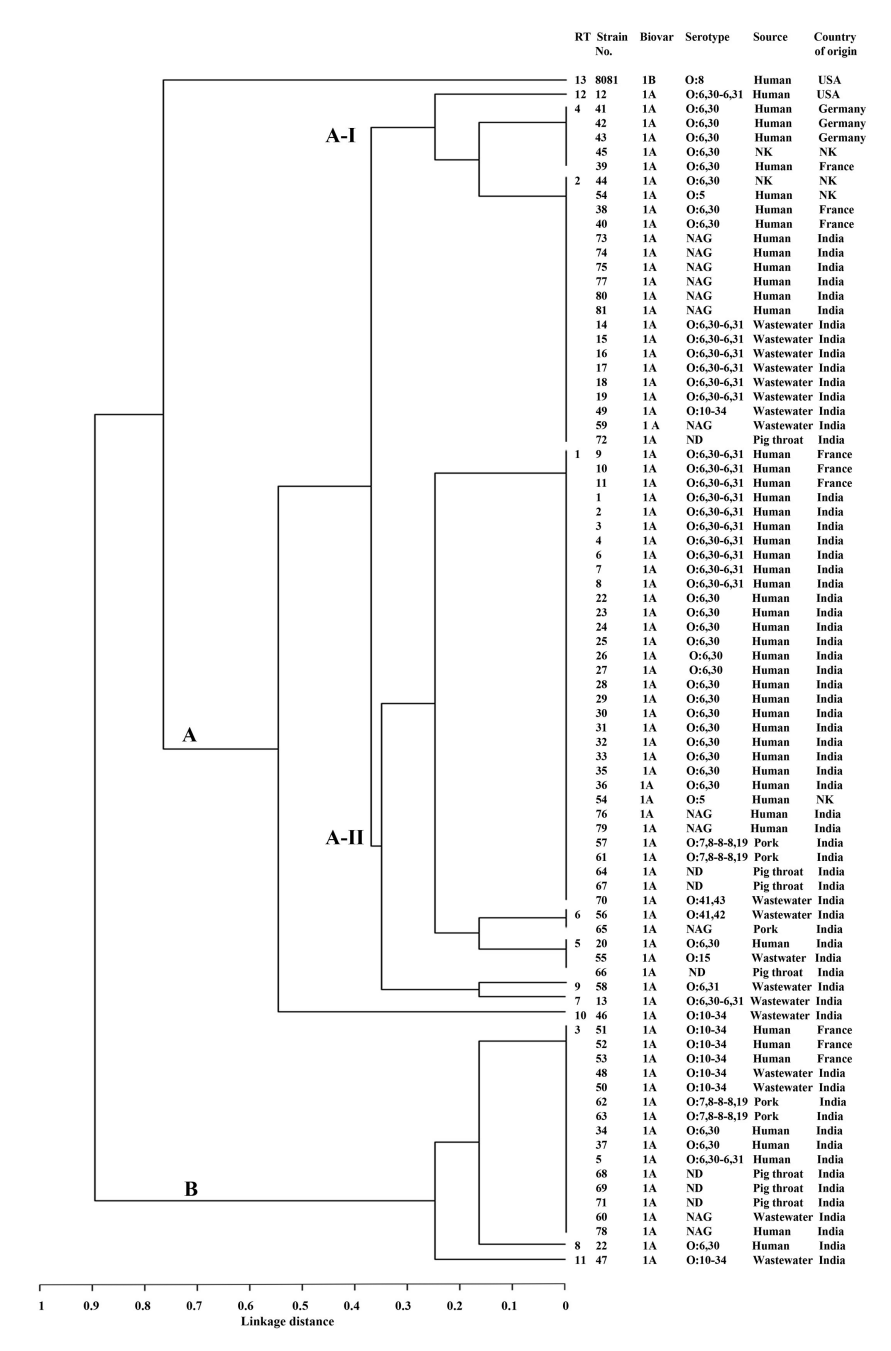
**Dendrogram showing relationships of *Y. enterocolitica *biovar 1A strains based on analysis of restriction types (RTs) generated by MLRT**. The dendrogram was constructed using UPGMA algorithm available in the START software package. NAG: non-agglutinable, ND: not determined, NK: not known.

The genetic relationships among strains analyzed by cluster analysis using UPGMA are shown in Figure 2. MLRT clustered biovar 1A strains into two clonal groups (A and B) while the reference strain (*Y. enterocolitica *8081, biovar 1B) formed a separate group, at the linkage distance of 0.78. The group A comprising most (64 of 81) of the isolates was represented by 9 different RTs. Within the group A, two subgroups were identified namely, A-I and A-II. In subgroup A-I, all wastewater serotype O:6,30-6,31 isolates, human NAG and European O:6,30 isolates were present. Subgroup A-II comprised of all clinical O:6,30-6,31 isolates, most clinical O:6,30 isolates, three pork and pig throat isolates each, and five wastewater isolates belonging to different serotypes. The most common RT, RT1 representing 31 isolates was present in this subgroup. The group B comprised of 15 isolates belonging to RT3 and a single isolate each of RT8 and RT11. Genotypically, this group was quite homogeneous despite belonging to different serotypes, sources and geographic origin.

**Figure 3:**
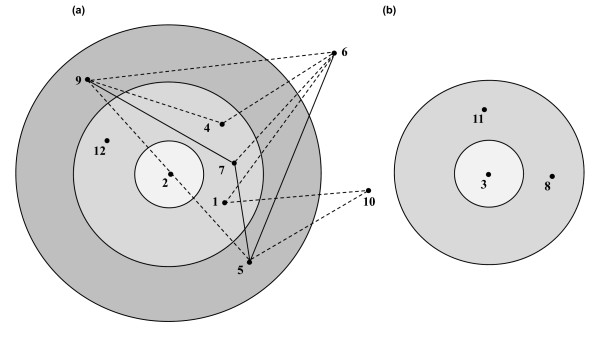
**Clonal complexes identified among 81 strains of *Y. enterocolitica *biovar 1A by BURST analysis of MLRT data**. a) Clonal complex A, b) Clonal complex B. Each number denotes a restriction type (RT; refer to Figure 2). Radial distribution shows divergent RTs. Ancestral RT is shown in the innermost circle. Single locus variants (SLV) are shown in the second circle and double locus variants (DLV) are represented in the outermost circle. Satellite RTs (RTs present outside the outermost circle) vary by more than two loci from the ancestral type. Lines indicate whether the RT is SLV (solid line) or DLV (dashed line).

The analysis of MLRT data by BURST program identified two clonal complexes (Figure 3) corresponding to the clonal groups identified above. The clonal complex A comprising 9 RTs (64 strains) revealed that wastewater serotype O:6,30-6,31 isolates represented by RT2 were present in the innermost circle as ancestral strains. The clinical serotype O:6,30-6,31 strains represented by RT1 and RT12 were present in the outer circle as single locus variants (Figure 3a) The double locus variants (RT5 and RT9) and the satellite RTs (RT6 and RT10) were represented by serotypes which are relatively not common. However, not much information could be inferred from clonal complex B (Figure 3b).

Sequencing of amplicons from representative strains confirmed the identity of the genes. Analysis of the sequences also confirmed the restriction patterns observed for each of the six genes. This is the first report on MLRT of *Y. enterocolitica*.

### Analysis of linkage disequilibrium and discriminatory indices

The frequency of recombination in natural populations can be estimated by calculating index of association (*I*_A_) between loci [35]. The results of the analysis of multilocus linkage disequilibrium in *Y. enterocolitica *are summarized in Table 4. The *I*_A _and *I*^S^_A _values for the 81 strains studied by MLEE were 0.613 and 0.128 respectively, which differed significantly (*p *< 0.001) from zero indicating that the strains were in linkage disequilibrium. Similarly, significant level of linkage disequilibrium was observed on analysis of MLRT data. The *I*_A _and *I*^S^_A _values were 3.357 and 0.672 respectively, and differed significantly (*p *< 0.001) from zero. Simpson's diversity index (DI) for MLEE and MLRT was 0.98 and 0.77 respectively.

**Table 4:** Multilocus linkage disequilibrium analysis of *Y. enterocolitica *biovar 1A strains

Method	Mean no. of alleles per locus	Mean genetic diversity (*H*)	*V*_E_*	*V*_O_*	*I*_A_	*I*^S^_A_	*P*†	95% critical value for *V*_O_
MLEE	7.5	0.566 ± 0.088	1.234	1.990	0.613	0.128	< 0.001	1.378
MLRT	3.2	0.441 ± 0.048	1.409	6.149	3.357	0.672	< 0.001	1.573

*: Calculated as described by Maynard Smith et al [35].

*V*_E_: expected variance, *V*_O_: observed variance, *I*_A_: Index of association, *I*^S^_A_: Standardized index of association.

†: Probability of observing *V*_O_/*V*_E _ratio as or more than that found in the original data calculated with 1,000 Monte Carlo randomizations.

## Discussion

Indexing allelic variations in sets of housekeeping genes provides a good measure of overall genetic heterogeneity in populations of microorganisms [21]. Methods based on this principle such as MLEE, MLRT and MLST (multilocus sequence typing) provide good insight into the genetic relationships among strains. In the present study, we used MLEE and MLRT to assess the genetic relationships among 81 strains of *Y. enterocolitica *biovar 1A isolated from clinical and non-clinical sources.

MLEE clustered *Y. enterocolitica *biovar 1A into four groups. A close analysis of data presented by Dolina and Peduzzi [23] who studied human, animal and aquatic strains of *Y. enterocolitica *isolated from Switzerland by MLEE, revealed that 51 biovar 1A strains clustered into two major groups, although minor clusters having one and six isolates each were also observed. Another study that used fluorescent amplified fragment length polymorphism (FAFLP) also clustered biovar 1A strains into two groups: one group comprised of biovar 1A strains; while a few biovar 1A strains clustered with atypical pathogenic biovars constituting the second group [39]. Further study by comparative genomic DNA microarray however showed that these biovar 1A strains constituted a single group [4]. Other studies using rep-PCR genotyping [17], 16S-23S IGS and *gyr*B RFLP [18], and MLVA [19] have also clustered biovar 1A strains into two clonal groups. MLEE revealed a total of 62 electrophoretic types (ETs) among 81 biovar 1A strains and showed high degree of discrimination (DI = 0.98). Studies of allelic variation by MLEE also revealed sufficient genetic diversity (*H *= 0.566) among strains of *Y. enterocolitica *biovar 1A. Similar genetic diversity was also reported in previous MLEE studies on *Y. enterocolitica *[22,23].

In the present study however, based on the number of distinct ETs generated, the clinical serotype O:6,30 and O:6,30-6,31 isolates were shown to be heterogeneous with mean genetic diversities (*H*) of 0.514 ± 0.112 and 0.442 ± 0.078 respectively. Previous studies in which other techniques namely rep-PCR [17], 16S-23S IGS and *gyr*B RFLP [18], and MLVA [19] were used to type these strains did not reveal this heterogeneity. Fearnley et al [39] also reported heterogeneity among serotype O:6,30 strains wherein seven AFLP types were identified among eight strains.

In the MLEE dendrogram, two ETs showed some pork and pig strains to be identical to the strains isolated from diarrheic human subjects suggesting that like pathogenic biovars [11,22,40], pigs may be the source of biovar 1A strains isolated from human patients. No such grouping of human and pork/pig isolates was evident from earlier studies [17,18]. However, this observation needs to be explored further by making use of a larger number of pig/pork isolates belonging to biovar 1A.

Multilocus restriction typing (MLRT) has recently been used to discern phylogenetic relationships among strains of *Streptococcus pneumoniae *[41], *Neisseria meningitidis *[28,42], *Burkholderia cepacia *[27,43], *Staphylococcus aureus *[44] and *Escherichia coli *[29]. MLRT has been reported to show good correlation with PFGE [27,29] and has been advocated as a cost effective alternative to MLST, which is relatively an expensive technique [28,42]. In the present study, MLRT divided 81 strains of *Y. enterocolitica *biovar 1A into 12 RTs based on a combination criteria of number of alleles and restriction patterns observed at each of the six loci examined. Cluster analysis of MLRT data revealed two clonal groups - A and B. The reference strain *Y. enterocolitica *8081 (biovar 1B) formed a distinct RT. Although MLRT profiles showed good reproducibility, the method failed to rival the discriminatory ability of MLEE. In the context of *Y. enterocolitica *biovar 1A, the discriminatory ability of MLRT (DI = 0.77) was lower than even rep-PCR (DI = 0.84) [17] and MLVA (DI = 0.87) [19].

Two clonal complexes were identified following BURST analysis of MLRT data. The primary clonal complex contained all but 3 RTs, representing 78% of the isolates. The other complex contained the remaining strains. The approach used in the BURST analysis specifically examines the relationships between closely related genotypes in the clonal complexes [45]. This analysis revealed that in the primary clonal complex, wastewater serotype O:6,30-6,31 isolates represented the ancestral strains while, clinical serotype O:6,30-6,31 strains occupied radial position as single locus variants. This observation corroborates the recent findings obtained from the study of VNTR loci which also suggested that the clinical serotype O:6,30-6,31 strains probably originated from the wastewater strains, by host adaptation and genetic change [19].

The analysis of linkage disequilibrium indicated clonal structure for *Y. enterocolitica *biovar 1A as values of *I*_A _and *I*^S^_A _were found to be significantly different from zero for both MLEE and MLRT data. Other genera, which have been reported to have clonal structure, include *Salmonella enterica *serovar Paratyphi B [46], *Mycobacterium *spp. [47], *Vibrio cholerae *[24] and *Pseudomonas stutzeri *[25].

Both MLEE and MLRT showed European strains to be more heterogeneous than the Indian strains. MLEE revealed that each of the 15 strains from France and Germany had distinct electrophoretic profiles indicating their heterogeneity. MLRT also revealed that the European strains, which displayed 5 RTs were more heterogeneous compared to Indian isolates. Genetic heterogeneity of European biovar 1A strains has been reported earlier using PFGE [48] and FAFLP [39]. A previous study using multilocus variable number tandem repeat analysis also identified 13 MLVA types among 15 European biovar 1A strains [19]. This suggests that European and Indian strains may constitute separate groups and might be evolving independently in two different settings. It would be interesting to explore these evolutionary aspects by comparative whole genome sequencing or multilocus sequence typing of Indian and European strains. It was also observed that strains with different serotypes (O antigen) types produced identical ETs or RTs and were closely related genetically. Also, in some cases, same O antigen was shared by strains that were different genotypically. These observations indicate O antigen switching in strains of *Y. enterocolitica *as suggested recently by MLST [49]. Such observations have however been reported in other bacteria also [24,41,50]. Thus, given the enormous discriminatory power of genotyping techniques such observations also emphasize the need to discuss threadbare, the question of suitability of widely used typing techniques like serotyping.

## Conclusion

More diversity was observed among clinical and non-clinical strains of *Y. enterocolitica *biovar 1A when MLEE was used. Sixty-two electrophoretic types were identified among 81 strains, which clustered into four distinct groups. MLRT identified 12 restriction types and was distinctly less discriminatory, clustering the strains into two groups. The BURST analysis of the MLRT data nevertheless provided newer insights into the probable evolution of clinical strains from those present in the aquatic environments.

## Authors' contributions

SM carried out the experimental part of the study. JSV conceived and supervised the work. Both authors participated in interpretation of data and preparation of the final manuscript.

## Supplementary Material

Additional file 1 **Representative restriction profiles of six genes of *Y. enterocolitica *biovar 1A**. (a) Malate dehydrogenase (*mdh*) digested with *Hae*III and *Rsa*I; (b) adenylate cyclase (*cya*) digested with *Hae*III and *Sau96*I; (c) gluamine synthetase (*gln*A) digested with *Sau96*I and *Bgl*I; (d) glucose-6-phosphate dehydrogenase (*zwf*) digested with *Rsa*I and *BstN*I; (e) isocitrate dehydrogenase (*icdA*) digested with *Rsa*I and *Hae*III; (f) glutamate dehydrogenase (*gdhA*) digested with *Hae*III and *Mse*I. Numbers above lanes represent the name of strain used to obtain the restriction pattern. Digestion products were compared to 100 bp (M) or 50 bp (M') DNA ladder. Click here for file
