# Supplementary figures and images for: Genetic relationships between clinical and non-clinical strains of Yersinia enterocolitica biovar 1A as revealed by multilocus enzyme electrophoresis and multilocus restriction typing
Source: BMC Microbiol. 2010 May 28;10:158. doi: 10.1186/1471-2180-10-158 (PMC2889952; doi:10.1186/1471-2180-10-158)

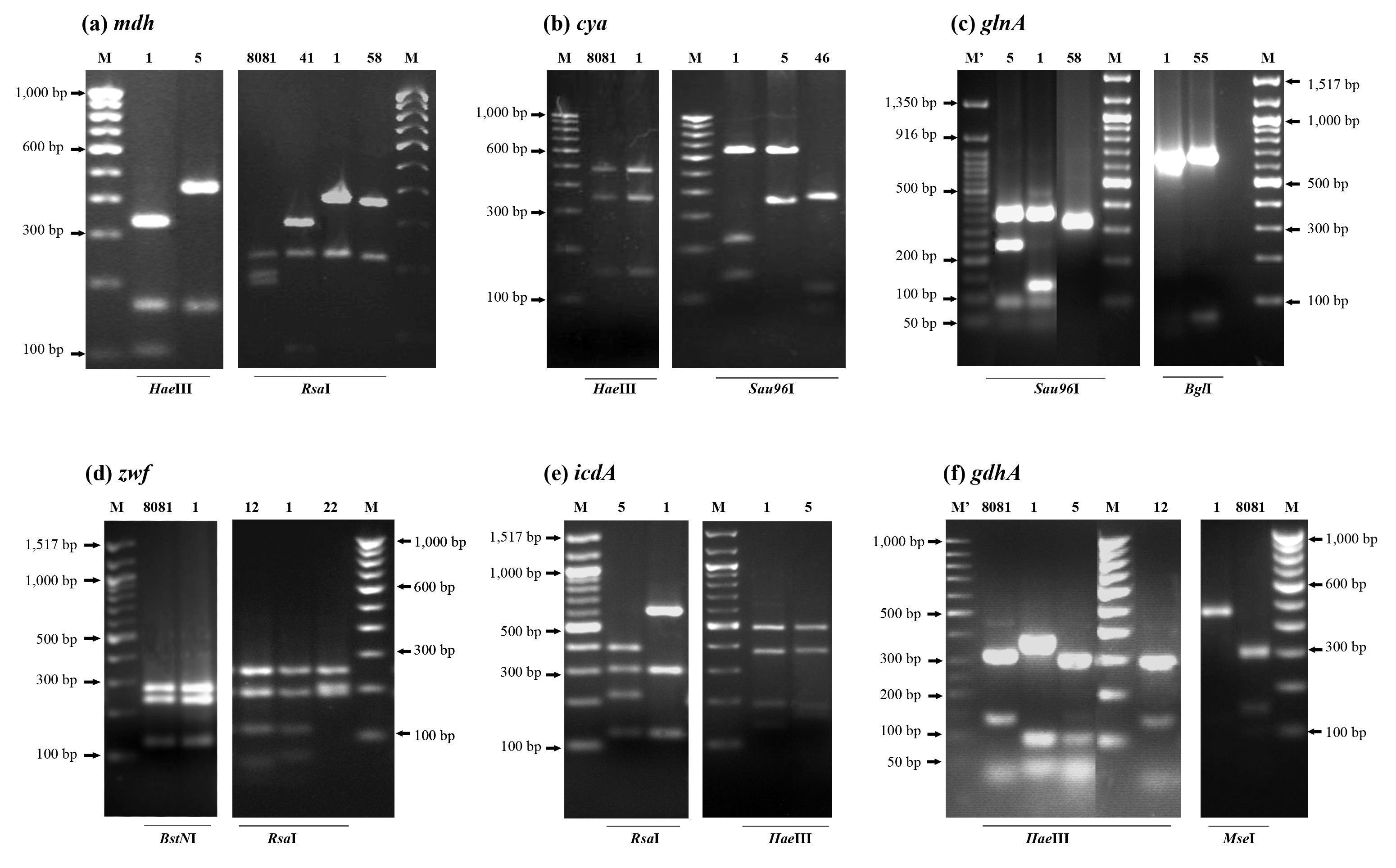

Supplement: Additional file 1 — Representative restriction profiles of six genes of Y. enterocolitica biovar 1A. (a) Malate dehydrogenase (mdh) digested with HaeIII and RsaI; (b) adenylate cyclase (cya) digested with HaeIII and Sau96I; (c) gluamine synthetase (glnA) digested with Sau96I and BglI; (d) glucose-6-phosphate dehydrogenase (zwf) digested with RsaI and BstNI; (e) isocitrate dehydrogenase (icdA) digested with RsaI and HaeIII; (f) glutamate dehydrogenase (gdhA) digested with HaeIII and MseI. Numbers above lanes represent the name of strain used to obtain the restriction pattern. Digestion products were compared to 100 bp (M) or 50 bp (M') DNA ladder. [file 1471-2180-10-158-S1.JPEG]
